# Plasminogen activator urokinase receptor as a diagnostic and prognostic biomarker in type 2 diabetic patients with cardiovascular disease

**DOI:** 10.34172/jcvtr.2023.32895

**Published:** 2023-09-23

**Authors:** Melak Saleh Mohammed, Hind Shakir Ahmed

**Affiliations:** Department of Chemistry, College of Education for Pure Science (Ibn Al-Haitham), University of Baghdad, Baghdad, Iraq

**Keywords:** Diabetes mellitus, Type 2 diabetes, Cardiovascular disease, Lipid indices, Plasminogen activator urokinase receptor

## Abstract

**Introduction:**

Cardiovascular diseases are the main cause of death among type 2 diabetic patients. Higher levels of plasminogen activator urokinase receptor have been found to predict morbidity and mortality across acute and chronic diseases in the common populace. This study aims to explore the role of serum plasminogen activator urokinase receptor levels as a cardiometabolic risk factor among type 2 diabetic Iraqi patients.

**Methods:**

Seventy type 2 diabetic patients (40 male and 30 female) (mean age: 46.20±7.56 years) participated in this study; 35 patients were with cardiovascular disease and 35 were without cardiovascular disease; their ages range was 40-55 years. In addition, 30 individuals who apparently healthy were selected as the control group.

**Results:**

There were significant increases (*P*<0.05) in glycemic and lipid profiles in diabetic patients with cardiovascular disease as compared to those without cardiovascular disease and control group. The present results reveal high levels of plasminogen activator urokinase receptor (2500.72±12.36 ρg/mL versus 2255.32±10.15 ρg/mL) with OR=1.80, 95%CI 1.2, and *P*=0.0001 in type 2 diabetic patients with and without cardiovascular disease respectively as compared to healthy control (229.00±14.48 ρg/mL).

**Conclusion:**

It has been concluded that serum plasminogen activator urokinase receptor showed higher levels among type 2 diabetic patients with cardiovascular disease, this revealed it’s critical role in cardiac disease. Therefore, it could be considered a more sensitive biomarker for the detection of cardiovascular events among type 2 diabetic patients who were at high-risk.

## Introduction

 Diabetes mellitus (DM) is a group of metabolic diseases that characterized by chronic hyperglycemia. It is initiated by higher blood glucose levels as a consequence of incapacity in pancreatic β-cells for producing sufficient insulin or ineffective insulin use by cells in the body, or both, caused by genetic or environmental influences.^1^

 Type 2 DM (T2DM) is manifested by disorder of carbohydrate, lipid and protein metabolism, usually it is related with insulin resistance (IR) in numerous tissues, although the particular mechanisms for these abnormalities remain to be determined.^2^

 People with T2DM are at high risk of micro- and macrovascular complications. Cardiovascular disease (CVD) is a main public health concern through the world. Diabetic patients have a 2-4 fold increased risk of progressing coronary artery disease (CAD), proving that T2DM is a distinctive risk feature for heart disease.^3^ In the preponderance of cases, the primary cause of CVD is atherosclerosis, which initiates when low-density lipoprotein (LDL) is oxidized and resulting in a flow of inflammatory cytokine production. The accumulation of oxidized LDL further damages the endothelial cells, leading to myocardial and cerebral ischemia. It has been suggested that chronic inflammation has a prospective role in atherosclerosis progression.^4^ Consequently, numerous study analysis data have examined the association between some of inflammatory biomarkers and CVD.^5^

 A potential blood biomarker reflecting this chronic inflammation, is plasminogen activator urokinase receptor (uPAR),^6^ also known as CD-87, it is a cysteine rich single chain glycoprotein with a relative molecular weight of 50-60 KD. In humans, uPAR is encoded by *PLAUR* on chromosome 19q13, comprising 52 amino acids at the N-terminus and 30 amino acids at the C-terminus, which is bounded by glycosyl phosphatidyl inositol anchor.^7^ The uPAR is cleaved and released from cells as a response to inflammation, producing soluble uPAR (suPAR), which can be considered as a pro-inflammatory marker in T2DM.^8^ The PA system is composed of uPA and its receptor (uPAR), tissue-type PA (tPA), plasminogen and its multiple receptors as well as three inhibitors (PAI-1, PAI-2, and protease nexin-1(PN-1)). These molecules are glycoproteins and found in most tissues and body fluids.^9^ The uPA–uPAR system is not only complicated in fibrinolysis, but it also controls cell proliferation, angiogenesis, adhesion and enrollment of inflammatory cells.^10^ Recently, suPAR has increased concern as a potential risk marker for T2DM, CVD, cancer and mortality in the widely populace.^11^ Higher serum suPAR levels have also been related with subclinical organ damage and CV complications, assisting to expect mortality causes in ischemic stroke.^12^

 The aims of this study is to examine the role of serum uPAR levels as a cardiometabolic risk factor among type 2 diabetic Iraqi patients.

## Materials and Methods

 This study was performed through October 2022 to February 2023 at Baghdad Teaching Hosptal/ Medical City-Baghdad. Seventy patients with T2DM were enrolled in the current study; 35 patients were with CVD and 35 without CVD. Moreover, 30 healthy individuals were selected as control group. Control subjects were collected as glycemic control when fasting serum glucose (FSG) < 100 mg/dL and glycated hemoglobin (HbA1c) < 5.7%.

 All the patients were enrolled from the outpatients’ departments of endocrinology and CVD at Baghdad Teaching Hospital in Baghdad, Iraq. A questionnaire was deliberated to contain age, height, weight, duration of DM and CVD, family history, smoking, and treatment. The diagnosis of DM was according to WHO criteria.^13^ In this criteria, a diagnosis of DM can be made by a FSG ≥ 126 mg/dL when HbA1c ≥ 6.5% is confirmed and T2DM patients were diagnosed by endocrinologist. While, CVD were diagnosed when two of the following three criteria were detected: typical symptoms, higher concentrations of cardiac enzymes (i.e., creatinine kinase above 5% of the total creatinine kinase, lactic dehydrogenase 1.5 times the upper limit of normal, or a troponin T level ≤ 2 ng/ml), or investigation changes on the electrocardiogram (ECG); they were examined by cardiologists.

###  Inclusion and exclusion criteria

**Figure 1:**
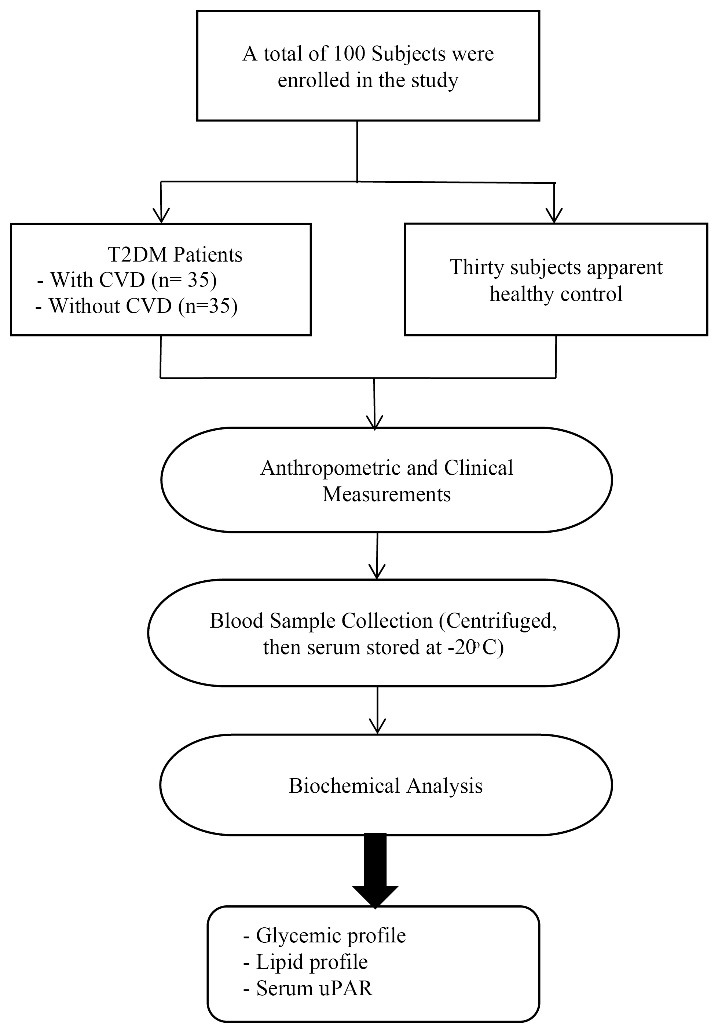
Flow-chart of the study

 The current study encompassed individuals who had T2DM with and without CVD; their ages ranged from 40-55 years. All diabetic patients were treated with anti-diabetic drugs. While, CVD patients were treated with aspirin, β-blocker, statin, angiotensin converting enzyme inhibitors, and angiotensin receptor blockers. Patients with T1DM, insulin users, a history of hepatic diseases, renal failure, thyroid disorders, autoimmune diseases, major chronic disorders, and pregnancy were excluded from this study. A flow chart of the study is illustrated in Figure 1.

 Anthropometric and clinical characteristics such as sex, age, weight, height, waist circumference (WC), waist to hip ratio (WHR), body mass index (BMI), systolic and diastolic blood pressure (SBP and DBP) for all participants were assessed.

 Blood samples were taken from each subjects in fasting state for laboratory investigation which included: FSG, total cholesterol (TC), triglyceride (TG), high density lipoprotein cholesterol (HDL-C), which were measured using biochemical automated analyzer (Cobas e411). The Bio-Rad VARIANT hemoglobin A1C employs automated and precise ion-exchange high-performance liquid chromatography (HPLC) principles to separate (HbA1c). Non HDL-C was designed by subtracting HDL-C from TC.^14^ Additionally, serum uPAR concentration was evaluated using sandwich enzyme immunoassay. The microtiter plate provided in this kit has been pre-coated with an antibody (Ab) specific to uPAR. Standards or samples were added to the appropriate microtiter plate wells then with a biotin-conjugated Ab specific to uPAR. Next, Avidin conjugated to horseradish peroxidase was added to each microplate well and incubated. After tetra methyl benzidine substrate solution was added, only those wells that contain uPAR, biotin-conjugated Ab and enzyme-conjugated avidin will exhibited a change in color. The enzyme-substrate reaction was terminated by the addition of sulphuric acid solution and the color change was measured spectrophotometrically at a wavelength of 450nm ± 10nm. The concentration of uPAR in the samples is then determined by comparing the optical density of the samples to the standard curve; Cat: ELK2317 and 2318 with sensitivity: 36 pg/mL and detection range: 78.13-5000 ρg/mL.

###  Statistical analysis

 The data were examined by the statistical package for social sciences (SPSS), version 25. All the results were expressed as means ± standard deviation (SD). Chi-Squared test was used for percentage values comparison. For the comparison of numerical variables between two groups with normally distributed data, the independent samples t-test was used. When comparing variables among more than two groups, the one-way analysis of variance (ANOVA) test was utilized. A *P-*value of less than 0.05 was reflected to be significant. Moreover, odd ratio (OR) and 95% confidence interval (95%CI) for uPAR levels were calculated.

## Results

 There were no significant differences in gender and age between the patients and control groups. While, there were significant increases* (P* = 0.0001) in other anthropometric and clinical features of diabetic patients as compared to the controls (Table 1).

**Table 1:** Anthropometric and clinical features of the study groups

**Parameters**	**Mean±SD**		***P***** value**
	**Patients** **(n=70)**	**Control** **(n=30)**	
Gender (Male/Female no, %)	40 (57.14%) 30 (42.86%)	20 (66.67%) 10 (33.33%)	0.306
Age (Years)	46.20 ± 7.56	41.53 ± 3.21	0.620
WC (cm)	106.18 ± 2.75	70.50 ± 1.32	0.0001
WHR	0.97 ± 0.03	0.72 ± 0.02	0.0001
BMI (kg/m2)	35.45 ± 2.45	21.52 ± 1.63	0.0001
SBP (mm Hg)	152.70 ± 3.20	113.00 ± 0.52	0.0001
DBP (mm Hg)	95.68 ± 2.14	78.50 ± 1.18	0.0001
Duration of DM (Years)	10.73 ± 4.52	-	-
Duration of CVD (Years)	5.43 ± 3.21	-	-
Family history (Yes/No)	62.8	0.30	0.0001
Smoking	50 (71.43%)	5 (16.67%)	0.0001

Data are expressed as a number (%) and mean ± SD; *p* < 0.05: Significant, *p* < 0.01: Highly significant, WC: waist circumference, WHR: waist to hip ratio, BMI: body mass index, SBP: systolic blood pressure, DBP: diastolic blood pressure.

Table 2 reveals significant increase (*P* < 0.05) in FSG, TC, TG, LDL-C, VLDL, and non HDL-C with a significant decrease (*P* = 0.001) in serum HDL-C in T2DM patients with CVD as compared to those without CVD and control group. Moreover, Table 2 shows high values of lipid ratios in T2DM with CVD as compared to those without CVD and healthy control.

**Table 2:** Glycemic and lipid profile in patients and control group

**Parameters**	**Mean±SD**			***P***** value**
	**T2DM with CVD** **(n=35)**	**T2DM** **(n=35)**	**Control** **(n=30)**	
FSG (mg/dL)	198.78 ± 10.12^a^	185.14 ± 8.73^b^	78.51 ± 4.21^c^	0.001
HbA1c (%)	14.65 ± 2.21^a^	9.50 ± 1.42^ab^	4.15 ± 0.40^c^	0.04
TC (mg/dL)	295.20 ± 10.15^a^	215.48 ± 8.12^b^	135.46 ± 8.10^c^	0.001
TG (mg/dL)	250.87 ± 12.40^a^	198.65 ± 10.70^b^	94.38 ± 15.61^c^	0.001
HDL-C (mg/dL)	35.21 ± 3.68^a^	42.27 ± 2.31^b^	68.62 ± 5.40^c^	0.001
LDL-C (mg/dL)	209.82 ± 4.15^a^	133.48 ± 3.25^b^	47.96 ± 2.43^c^	0.001
VLDL (mg/dL)	50.17 ± 2.42^a^	39.73 ± 2.16^b^	18.88 ± 3.12^c^	0.001
Non HDL-C (mg/dL)	260.08 ± 6.43^a^	173.20 ± 5.84^b^	66.84 ± 3.90^c^	0.001
TC/HDL-C ratio	8.38 ± 2.78^a^	5.00 ± 3.48^ab^	1.70 ± 0.52^bc^	0.04
LDL-C/HDL-C ratio	5.95 ± 1.12^a^	3.14 ± 1.38^ab^	0.65 ± 2.34^c^	0.03
TG/HDL-C ratio	7.17 ± 3.35^a^	4.68 ± 2.31^ab^	1.37 ± 1.44^bc^	0.03

Data are expressed as mean ± SD. Same letters indicate no significant changes and different letters indicate significant changes, *P* < 0.05: Significant, *P* < 0.01: Highly significant, FSG: fasting serum glucose, HbA1c: glycated hemoglobin, TC: total cholesterol, TG: triglyceride, HDL-C: high density lipoprotein cholesterol, LDL-C: low density lipoprotein cholesterol, VLDL: very low density lipoprotein.

Table 3 explains serum uPAR levels and OR for patients and control groups. SerumuPAR levels were significantly increased (*P* = 0.0001) with OR = 1.80 and 95%CI = 1.2 in diabetic patients as compared to the controls. Moreover, a highly significant increase (*P* = 0.0001) were found in serum uPAR level in T2DM with CVD as compared to those without CVD.

**Table 3:** Serum uPAR levels with OR for the studied groups

**Parameters**	**Mean±SD**			**OR**	**95%CI**	***P***** value**
	**T2DM with CVD** **(n=35)**	**T2DM ** **(n=35)**	**Control** **(n=30)**			
uPAR (ρg/mL)	2500.72 ± 12.36^a^	2255.32 ± 10.15^b^	229.00 ± 14.48^c^	1.80	1.2	0.0001

Data are expressed as mean ± SD. Different letters indicate significant differences, *P* < 0.01: Highly significant, OR; odd ratio, CI: confidence interval, uPAR: plasminogen activator urokinase receptor.

**Figure 2:**
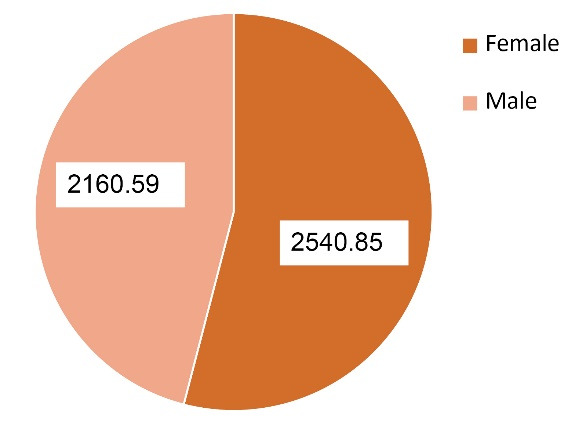
Gender distribution of uPAR levels in diabetic patients groups

Table 4 and Figure 2 revealed that female patients had significant increase (*P* = 0.0001) in serum uPAR levels as compared to the male patients.

**Table 4:** Effect of gender on uPAR levels in diabetic patients groups

**Parameters**	**Mean ± SD**		***P***** value**
	**Female** **(n=30)**	**Male** **(n=40)**	
uPAR (ρg/mL)	2540.85 ± 10.15	2160.59 ± 14.48	0.0001

Data are expressed as mean ± SD, *P* < 0.01: Highly significant, uPAR: plasminogen activator urokinase receptor.

 There were significant positive correlations between serum uPAR levels and age, WC, WHR, BMI, SBP, DBP, FSG, HbA1c, TC, TAG, VLDL, LDL-C, and non HDL-C in diabetic patients groups (Table 5).

**Table 5:** Correlation coefficient of serum uPAR with study parameters in T2DM and T2DM with CVD

**Parameters \ uPAR (ρg/mL)**	**T2DM with CVD** **(n=35)**		**T2DM** **(n=35)**	
	**r**	*** p***	***r***	***p***
Age (Years)	0.938**	< 0.001	0.961**	< 0.001
WC (cm)	0.956**	< 0.001	0.964**	< 0.001
WHR	0.297*	0.036	0.560**	< 0.001
BMI (kg/m^2^)	0.152	0.294	0.649**	< 0.001
SBP (mm Hg)	0.977**	< 0.001	0.948**	< 0.001
DBP (mm Hg)	0.910**	< 0.001	0.948**	< 0.001
FSG (mg/dL)	0.955**	< 0.001	0.621**	< 0.001
HbA1c (%)	0.936**	< 0.001	0.966**	< 0.001
TC (mg/dL)	0.944**	< 0.001	0.965**	< 0.001
TAG (mg/dL)	0.923**	< 0.001	0.989**	< 0.001
HDL-C (mg/dL)	-0.953**	< 0.001	-0.970**	< 0.001
VLDL (mg/dL)	0.923**	< 0.001	0.989**	< 0.001
LDL-C (mg/dL)	0.281*	0.048	0.969**	< 0.001
Non HDL-C (mg/dL)	0.965**	< 0.001	0.973**	< 0.001

*P < 0.05: Significant, **P < 0.001: Highly significant, WC: waist circumference, WHR: waist to hip ratio, BMI: body mass index, SBP: systolic blood pressure, DBP: diastolic blood pressure, FSG: fasting serum glucose, HbA1c: glycated hemoglobin, TC: total cholesterol, TG: triglyceride, HDL-C: high density lipoprotein cholesterol, LDL-C: low density lipoprotein cholesterol, VLDL: very low density lipoprotein, uPAR: plasminogen activator urokinase receptor.

## Discussion

 Cardiovascular disease represent a health concern expected to be an atherosclerotic cause and denotes primary cause of death among diabetics. Various assumptions from epidemiological data analysis have revealed the significance of CV risk factors among diabetic patients. Though diabetes considers a progressive risk for health, there are simultaneous illnesses with DM, i.e., dyslipidemia, hypertension, and obesity, and that denote major risk causes for atherosclerosis.^15^ Age-associated variations in CV events and the clinical consequences of CV ageing are well recognized.^16^ From this study, concluded that smoker patients represents 71.43%, which revealed serious health consequences for the smoker in terms of its adverse and high effect on the body fat levels, causing dyslipidemia which leads to atherosclerosis and CVD.^17^

 The current outcomes revealed that diabetic patients with CVD had family histories of metabolic complications, also had higher anthropometric and clinical features, i.e., WC, WHR, BMI, SBP, and DBP. Moreover, the current study suggested considerable increases in FSG and HbA1c among diabetic patients with and without CVD comparing to healthy control.These consequences are due to hyperglycemia, which is the main distinctive feature of DM. Blood glucose is toughly controlled by two leading processes: insulin secretion and insulin action on main tissues, i.e., skeletal muscle, liver, and adipose tissue. Type 2 DM is frequently related with obesity and IR characterized by hyperinsulinemia.^18^ Although its inadequacy among patients with hemoglobinopathies, HbA1c is the gold standard for defining glycemic control among diabetic patients. It is used to give idea on glycemic control.^19^ In the same content, diabetic patients in this study had raised value of HbA1c, which is in accordance with previous study.^20^

 Dyslipidemia is main irregularity in DM, and it is characterized by higher TG levels, diminished HDL-C concentrations and higher or normal LDL-C levels. One study showed that TG/HDL-C ratio is interrelated with IR, and a higher value of it suggests the triggered secretion function of islet β-cells.^21^ Further study showed that obesity can lead to IR and promote abnormalities in glucose metabolism, that finally leads to DM.^22^ Consequently, the combination of obesity with dyslipidemia can raise the risk of DM. On the one hand, the insulin-mediated activation of the phosphoinositide 3-kinase pathway is decreased, while activation of the extracellular signal-regulated protein kinase 1/2 and the production of endothelin-1 by insulin are normal.^23^ On the other hand, it has been found that cholesterol homeostasis is related with insulin secretory, and dyslipidemia causes defects in insulin secretion and alterations of glucose metabolism. Also, higher LDL-C and diminished HDL-C levels, as risk features for β-cell dysfunction, increase the bio-disposal of LDL-C in the pancreatic cell metabolism to have a cytotoxic influence; they may also increase β-cell apoptosis, with an influence of IR on DM, thereby decreasing insulin sensitivity and impairing pancreatic β-cell function.^24^ Dyslipidemia is triggered by the alteration in lipoprotein activity, and it can also progress into IR and ultimately lead to DM. Furthermore, in obese subjects, the higher free fatty acids that inter to the liver leads to the accumulation of TGs andrises LDL-C synthesis.^25^

 The atherogenic indices (ratios), which is reflected by LDL-C/HDL-C and TC/HDL-C ratios is more prognostic as constituents and indicator of CV risk as compared to the traditional lipid factors.^26^ The LDL-C/HDL-C ratio has been revealed to be the most related index for CV risk rendering to previous study.^27^ The LDL particles size index (TG/HDL-C) ratio was significantly elevated in diabetic patients. Hence, it could be used effectively as a further index for assessment of CV risk, which is in agreement with previous study.^28^ Earlier data documented that the risk values of LDL-C/HDL-C ratio are > 3.5 for men and > 3.0 for women, while the goal values for both gender are 3.0 and 2.5, respectively.^29^

 A chronic inflammatory state mediated by mechanical and humoral influences, is frequently designated in ageing related CVD. This suggests that suPAR is a prospective indicator for heart disease. It is suggested as a biomarker of acute and chronic organ damage. However, it was considered as a biomarker of low-grade inflammation, which is detected in CVD, i.e, CAD or stroke.^30^ Consequently, higher levels of suPAR is noticed in endothelium dysfunction, vascular stiffness and lead to atherosclerosis. It has been examined varied levels of suPAR via different study analysis as a biomarker or risk factor of severity in stroke and heart attack, but these outcomes are indistinct. The present results confirm that higher levels of suPAR are detected in diabetic patients with CVD. Persson et al described the correlation between serum suPAR and the occurrence of stroke and CAD.^31^ Edsfeldt et al study revealed higher concentrations of suPAR in patients with symptomatic atherosclerosis, compared to asymptomatic patients.^32^ Systematic alterations in cardiac and vascular structure and function lead to heart failure (HF) and cerebrocardiovascular events. The presence of low-grade chronic inflammation processes, mainly on the endothelial level, results in subclinical organ damage leading to diabetes, HF, malignancy and inflammatory systemic diseases. This may be clarified by the fact that raised suPAR levels are not definite for atherosclerosis only, but also reveal the activation of various inflammatory and proinflammatory cells, chemokines and cytokines. So, it is supposed that proinflammatory cytokines trigger the release of suPAR from activated monocytes, neutrophils, and endothelial cells. This inflammatory progression is maintained by suPAR because it acts as a chemotactic agent that stimulates enrollment of immune cells to sites of acute inflammation.^33^ Additionally, suPAR meets critical applications for a biomarker as it is frequently stable in plasma, unlike numerous biomarkers, free of circadian variations and stable during periods of acute stress. It was previously proved that suPAR level is elevated in individuals with the history of CVD and their complications. It also predicts adverse cardiac events, especially in patients after the first episode. This may be due to the infectious complications of stroke, which are frequent among patients with stroke. Hence, both suPAR and advanced ECG were found valuable investigative tools for categorizing diabetic patients at risk of prospect clinical cardiac disease.^34^

 The uPAR and uPA have a role in the pathogenesis of vascular diseases, which is accompanied by inflammation. At the cell surface, uPAR can be cleaved by its ligand uPA or further proteases, thus releasing suPAR to the bloodstream or other body fluids. Atherosclerotic plaque causes an increase of macrophages and leads to the release of uPAR from their surface.The specific inflammatory mediators revealed the expression of uPAR alongside the release of suPAR *in vitro* and *in vivo* comprise lipopolysaccharide (LPS), which rises the mRNA expression of uPAR *in vitro*, and triggers the release of suPAR. Injection of LPS in healthy individuals has also been increased the suPAR levels in blood, also increased the expression of uPAR on circulating monocytes.^35^ Release of suPAR from immune cells is related with the inflammatory status; so, the blood suPAR level is revealed an person’s level of inflammation and immune activation. The median suPAR level is around 2 ng/mL, and women usually have greater suPAR than men. Though, suPAR appears to rise with age in men as compared to women, and no changes among the both gender at age ≥ 74 years,^36^ which is in agreement with the current outcomes. These differences between gender may be due to ethnic variances, diet, lifestyle, demographic factors, analysis methods, metabolic and hormonal changes are affected on uPAR value.^37^ Also, drugs that are identified to influence lipid metabolism such as lipid-lowering drugs, β-blockers, or diuretics might be considered.^38^ Also, suPAR levels are greater in serum than in plasma in humans. The suPAR concentration is influenced by numerous elements, comprising heredities, lifestyle, acute- and chronic illness, but the contributions of these influences to suPAR levels has not been completely understood. Additionally, the same factors also affect the tissue uPAR expression. The suPAR is removed from the circulation by cardiac clearance and renal excretion.^39^ Elevated suPAR concentrations are considerably related with deterioration in renal function, and as a consequence of reduced filtration in patients on dialysis, so high levels of suPAR could be released. Nevertheless, high levels of suPAR might be due to the deterioration in glomerular filtration rates, indicating that it is related with kidney function only. So, uPAR has several vital roles in the inflammatory response, comprising cell migration, proliferation, invasion, phagocytosis, vasodilation, in addition to release of cytokines and chemokines.^40^

 The current study has many limitations. The study was cross-sectional, hence it is unable to determine causality. The sample size is relatively small and contribution was voluntary, and inclusion of subjects who were mostly more concerned in their health. Even so, the true association between uPAR and CVD may be overestimated.

 The uPAR has many domains while, the assay used in this work might not distinguish between the forms of uPAR. Hence, this study is incapable to detect associations of different these subtypes to CVD.

## Conclusion

 It has been concluded that serum uPAR showed higher levels among type 2 diabetic patients with CVD, this revealed it’s critical role in cardiac disease. Therefore, it could be considered as more sensitive biomarker for the detection of CV events among type 2 diabetic patients who were at high-risk.

## Acknowledgements

 The authors thank the staff of Baghdad Teaching Hospital/ Medical City-Baghdad, Iraq for their cooperation during the work. Also, more appreciation for patients who were participated in this study.

## Competing Interests

 The authors declared no competing of interest.

## Ethical Approval

 The Institutional Scientific Committee at the University of Baghdad approved this study according to Declaration of Helsinki for humans studies (Consent number: 4737 at 13/9/2022).

## Funding

 This research received no specific grant from any funding agency in the public, commercial, or not-for-profit sectors.
